# Preserving togetherness or ensuring safety? The dilemma of where to live and receive dementia care

**DOI:** 10.1186/s12877-026-07884-y

**Published:** 2026-06-24

**Authors:** Stina Engelheart, Lisa Spang

**Affiliations:** 1 School of Medical Sciences, Örebro University, Örebro, Sweden; 2 Social Services Administration, Örebro Municipality, Örebro, Sweden; 3 School of Health Sciences, Örebro University, Fakultetsgatan 1, Örebro, SE-701 82 Sweden

**Keywords:** Ageing, Case-study, Qualitative method, Residential care

## Abstract

**Background:**

As ageing populations become increasingly culturally diverse, the need for responsive dementia care grows. However, there remains limited knowledge about how older immigrants experience ageing in general and, specifically, dementia within a foreign cultural and societal context. This affects both the person with dementia and their caregivers. The aim of this study was to explore how older immigrants with dementia (and their families) wish to live their lives and receive care and support throughout the progression of the disease.

**Methods:**

A qualitative case study design was used. Older immigrants with dementia and their family members were interviewed about 1) their views on engaging with formal dementia care and 2) their thoughts regarding admission to residential care. Two contrasting cases formed the basis of the analysis.

**Results:**

The overarching theme, Residential Care as the End Point of Support Strategies, captured shared experiences across both cases despite differing circumstances. The first case concerned a couple living together, where the husband had dementia; this was represented in the theme Preserving Togetherness in the Face of Decline. The second case involved a couple in which the husband had dementia with behavioural and psychological symptoms; this was reflected in the theme Safety Over Togetherness. Across both cases, the transition from home to residential care was described as an existential threshold shaped by institutional routines, uncertainty about staff competence, cultural and linguistic mismatches, and resistance to relocation.

**Conclusion:**

The described barriers contributed to fears of losing personal identity, daily habits, and (culturally) meaningful ways of being. The findings highlight the need for stakeholders and policymakers to address these vulnerabilities when developing healthcare and social services for immigrants living with dementia.

**Supplementary Information:**

The online version contains supplementary material available at 10.1186/s12877-026-07884-y.

## Background

The prevalence of dementia is an escalating global public health challenge that profoundly affects individuals, families, and societies [1]. As populations become increasingly multicultural, the number of people living with dementia and a migration background is also increasing [2]. Further, developing dementia in a foreign country could entail confronting multiple layers of vulnerability and insecurity, both for the person with dementia as well as for the family. This may include language barriers which can delay or hinder both diagnosis and treatment, and cultural differences that may influence how symptoms are interpreted. Several review studies [3–5] have shown that both formal and informal caregivers are affected in the long term according to what care and support is sought and how it could be provided. Previous research also indicates that immigrants often are diagnosed with dementia at a later stage, which can mean reduced access to specialised care and greater social isolation [5]. However, a review by Lillekroken et al. [6] emphasises the importance of recognising that these experiences (of family caregiver) are not homogeneous, as they are shaped by factors such as reasons for migration, length of stay in the host country, level of education, socioeconomic status, and access to culturally appropriate care.

Individuals with dementia require ongoing care and support in daily life activities as the disease progresses, often provided by family members acting as informal caregivers [7]. The need for care also increases as the disease progresses. In early stages of the disease the persons often struggle to manage their cognitive symptoms, adapt to behavioural changes, and conduct routine daily activities independently [8]. Without consistent support, maintaining a sense of normalcy and personal dignity can become increasingly difficult. The informal caregivers are frequently involved in navigating and managing complex medical needs, unpredictable behaviours, and making tough decisions about care and safety of their family member [9]. The responsibility of supporting a family member with dementia can be emotionally and physically demanding for the family caregiver and can therefore be associated with heightened stress, emotional strain, and a sense of burden, which can impact the caregiver’s own health and wellbeing over time [10]. Despite these challenges, family members continue to provide care out of love, duty, or necessity, often with limited access to professional support or respite services [11]. Caring for a family member, instead of receiving formal care or admission to residential care, can be deeply rooted in the culture and be interpreted as a natural part of life [12]. A combination of a lack of health literacy of dementia in the individuals themselves and their family, e.g. a lack of knowledge of the disease of dementia as well as the possibilities of support and care, affects the willingness or possibility of applying for formal support [3, 13]. Additionally, Nielsen et al. [14] have shown that a lack of insight, cultural competence and adaptation within existing formal care makes fewer next of kin of older immigrants consider admission to residential care with all-day care and support.

Recognising and addressing the needs of both individuals with dementia and their caregivers is essential to ensuring compassionate and sustainable care within the community. However, the increasing cultural diversity in many countries also needs to be addressed. There is a significant gap in scientific knowledge regarding how older immigrants experience ageing and the progression of dementia in a foreign cultural and societal context. Considering the unique challenges of this population which are shaped by migration history, language barriers, cultural norms, and differing expectations of care, this knowledge gap has implications for the development of culturally responsive dementia care and support systems in Sweden, as well as in other countries. The lack of understanding of experiences and preferences of care of older immigrants increases the risk that existing services may not adequately meet their needs or respect their cultural identities. Further research is essential to develop policies and practices that promote equitable access to high quality care for all older adults, regardless of cultural or linguistic background. Therefore, the aim of this study was to explore how older immigrants with dementia wish to live their lives and receive care and support throughout the dementia progression.

## Methods

A qualitative case study design was chosen [15] to enable an in-depth exploration of how older immigrants, now living in Sweden, think about taking part in official Swedish dementia care services, specifically residential care. This methodological approach allows for a nuanced understanding of how cultural background influences perceptions of support, adaptation, and the relevance of available care and support options. Also, findings in previous research on immigrants with dementia proposed that qualitative studies, instead of surveys, would increase participation in this target group for research [16]. To analyse the data, a reflexive thematic analysis was employed [17–19], as it offers a flexible and theoretically grounded approach for interpreting complex, context-dependent experiences from two cases. These two cases illustrate different but similar experiences of an increasing need for care in Sweden. Further, this study followed the guidelines of qualitative research according to COREQ [20]. Ethical approval was obtained from the Swedish Ethical Review Authority, Dnr: 2024-01131-01.

### Data collection

This study recruited older immigrants (65 years old or older) with dementia to interview them (and/or their families) about their thoughts about receiving official dementia care and about the factors that are important when (or if) the person is admitted to residential care. Both the individuals themselves and their family members were considered for recruitment into the study.

Potential participants were identified through municipality home care units, a memory clinic, organisations, associations, and dedicated individuals within various cultural communities. However, the recruitment process lasted about twelve months (2024–2025), and only two couples gave their consent to participate, each including one person with dementia (husband) and one partner (wife). Healthcare staff at these facilities have extensive experience working with individuals with cognitive impairment and determined that the person interviewed individually possessed the cognitive ability to speak for themselves, a view shared by the interviewer. All four persons signed a formal declaration of consent to participate in the interview. Reasons for declining participation included cultural perceptions of dementia; as it was explained to us that in some communities dementia is not recognised as a disease, but rather as a natural part of ageing, also described as forgetfulness by Mazaheri et al. [21]. Also, some individuals had received a formal diagnosis, but in some cultures, family members preferred to protect the person and were not interested in sharing their experiences with health care personal or with researchers.

The first authors interviewed the two couples. One couple was interviewed together in their home, and the other couple separately, one at home and the other in a separate room in a public library. The interviews were semi-structured, where the interviewer used an interview guide (See supplementary file) including questions such as “Tell me your thoughts about receiving dementia care,” and “Have you received information about what kind of support admission to residential care could offer?”. All four participants shared experiences about their daily struggles related to dementia symptoms, how they had tried various kinds of support and about their thoughts about admission to residential care. The interviews were audio-recorded and then manually transcribed. For more information on the two cases, see Table 1, Demographic characteristics of the participants.

**Table 1:** Demographic characteristics of the two cases (both person with dementia and the partner)

	Case 1	Case 2
Nationality	Bosnian	France/Egyptian
Language	Croatia/ Bosnia	French/Arabic
Living situation	Living together Town house Rural	Living apart Apartment / Sheltered housing for woman Urban
Support from municipality	Social activities for people with dementia Organised support for relatives	Contact with cognitive support team in municipality Women’s shelter

Information regarding the participants’ ages was not collected

### Data analysis

To analyse the interviews, reflexive thematic analysis was chosen as it allowed the authors to be involved in the analysis process, thereby fostering a richer and more nuanced reading of participants’ narratives. This analytic approach is particularly well-suited to the aim of the study, to capture culturally embedded understandings of how the participants wanted to receive dementia care now and in the future. Both authors conducted the analyses, identifying codes separately in both cases (couples). They then met to discuss their code similarities and how they interpreted the data: similarly, or differently. Both authors are female researchers with diverse backgrounds. The first author is a registered dietician with limited experience of qualitative research but extensive experience of meeting and talking to people with the same life situation as the study participants. The last author is an occupational therapist with experience of qualitative research and of working with older adults with various kinds of needs in daily life. These differences influenced their interpretation of the data. Discussions were held on similarities between the two cases interviewed in this study, and common threads that matched the purpose were found. Table 2 illustrates the combined subthemes that represent the two themes and the overarching main theme.

**Table 2:** Presentation of the combined subthemes that represent the two themes and the overarching main theme

Residential Care as the End Point of Support Strategies	
Preserving Togetherness in the Face of Decline	Safety Over Togetherness
* Crossing the Threshold: From Home to Facility*	
* Barriers to Admission for Residential care*	

## Results

The main theme was Residential Care as the End Point of Support Strategies and reflects the combined experience despite different living conditions and experiences. The main theme is based on descriptions from the two cases; 1) A couple that lives together, where one of them (husband) has dementia. This case is described in the theme Preserving Togetherness in the Face of Decline and 2) A couple that lives together where one of them (husband) has dementia with behavioural and psychological symptoms of dementia (BPSD) including aggression and paranoia. These symptoms led to the fact that the partner without dementia (wife) in period(s) lived in a women’s shelter. This case is described in theme Safety Over Togetherness.

The two cases have commonalities in the experience of one person living together with and caring for someone with dementia, which leads to the thought of need for residential care. However, the two cases reflect different living situations in relation to the stage and symptoms of dementia, resulting in different demanding experiences in everyday life. The husbands with dementia had limited ability to answer questions and follow the conversations in the interview situations. Therefore, the results are mostly based on information from the wives. Their experiences are represented in common subthemes: Crossing the Threshold: From Home to Facility and Barriers to Admission for Residential care.

### Preserving togetherness in the face of decline

#### Crossing the threshold: from home to facility

The partner without dementia (the wife) revealed the emotional complexity and practical considerations involved in transitioning from their current home to admission to residential care. The wife speaks openly about the inevitability of this move yet emphasises how difficult and painful the decision will be for both of them. She describes it as something they have discussed a lot, but which must be approached gradually and with care.

*“Well, uh, we agree that we will take it step by step. It’s not an easy decision to move [to another housing]. Not for him, nor for me… (becomes tearful) but we know it will happen eventually.”*

The wife actively sought official information about care and support from various sources e.g., care managers, support centres, and online platforms and she felt confident about who to contact when the time came. She also had experience of working in elderly care herself, which gave her insight into the system, but this also fuels her scepticism and concern about institutional routines that may override personal autonomy, and of the difficulties of being an older person with another cultural background in need of care due to cognitive decline.

Furthermore, she emphasised the importance of maintaining one’s identity in combination with new habits and personal traits due to the progression of dementia. She described how her husband’s habits had changed significantly, for instance by switching between Swedish and his native language. Also, his habits during the day have shifted towards more often being awake during the night, drinking coffee and reading books in his armchair. His appetite and meal habits have also changed, as he does not always eat at regular times, and his appetite for meals could differ throughout the day. The wife described that he comes into the kitchen and nibbles on food while she is cooking, especially when the smell of familiar dishes filled the apartment. She also had noted that he needs more seasoning in the food to make it palatable for him. She also describes that her husband has changed in relation to what foods he likes or dislikes; now he can eat foods he did not like before, with a good appetite, and vice versa. She expressed acceptance of these changes in her husband and described her ways of adapting to these changes. However, for her, person-centred care meant allowing him to make his choices and live according to his preferences, and this would be most important when (if) admission to residential care is necessary.

*“He gets up in the middle of the night, drinks coffee, and sits in his armchair reading books. He does it for hours, and then he is calm… sometimes he is awake during the night but sleeps all day. Sometimes he wears his pyjamas all day long, but I let him be.”*

Contrary to her adaptations due to her husband’s symptoms, she described her experiences from working in elderly care, that this is not possible in residential care, where residents are expected to conform to institutional routines. These experiences worried her, as it was not the future she wanted for her husband. Her goal was to continue caring for him in their shared home for as long as possible. She describes the changes and behaviours as details, but she was also clear that this could be of most importance for an individual. She also mentioned that she was accustomed to caregiving, not only through her professional background but also from having cared for her own mother at home until the end of her life.

#### Barriers to admission to residential care

Based on previous experiences working with residential care, the wife related to that manager declined staff competence and adequate training, and that is the staff had an overwhelming workload. She talked about how she remembers situations where the staff were burdened with non-care-related tasks such as laundry, documentation, and cleaning, leaving limited time for meaningful interaction with residents. These conditions contribute to her lack of trust in the system’s ability to provide compassionate and individualised care for her husband.

She expressed strong preferences about being respected in a care facility: maintaining lifelong habits like taking a shower in the evening and not in the morning, eating meals privately rather than together with other residents in a communal dinner room, and choosing whether to participate in activities or not. She was particularly concerned about preserving dignity, for instance regarding receiving care of intimate hygiene from the opposite sex or a much younger person and the right to make decisions without being rushed or overruled.

*“And don’t drag me into different activities just because someone happens to be there. I decide for myself whether it’s something I’m interested in or not at all. And no one should knock on my door at 7:30 p.m. asking*,* ‘Are you going to bed?’ Absolutely not!”*

Cultural and linguistic factors add an additional layer of complexity to the expressed importance when (if) moving to residential care. As her husband’s dementia progresses, he increasingly reverts to his native language, which raises concerns about communication and emotional safety in a facility where staff may not understand him. The wife fears that misunderstandings could lead to distress or inappropriate care responses. The physical environment of care facilities also matters deeply. She raises the issue that some care units for people with dementia are not placed on the ground floor of a building and are therefore without direct contact to outdoor space. The closeness to outdoor facilities was raised as important for her husband’s quality of life, despite his dementia. She argues that such design choices ignore the needs of people with dementia who benefit from nature, movement, closeness to physical activity and familiar surroundings.

### Safety over togetherness

#### Crossing the threshold: from home to facility

The husband with BPSD did not himself raise any issues (either pro or con) related to admission to residential care. In the interview situation he consistently avoided answering any related questions, by saying that he would like to stay in his apartment as long as possible, but that he has heard about housing “when I am totally lost in my dementia and don’t remember my family, then I need support”. However, the wife described that her husband expressed strong resistance to the idea of relocating to a residential care setting and that he did not want to talk about it. This reluctance, she thought, was tied to a sense of independence and status, with moving being perceived as a symbol of losing control and dignity.

The wife described how her husband has become jealous and violent due to his BPSD and how leaving home (periodically for a women’s shelter) was not simply a physical move, but that it represents a profound shift involving identity, security, and relationships. She also describes her husband as being jealous and violent because of his dementia symptoms, and how his resistance to moving had led to conflict and emotional strain, particularly when the PBSD progressed and home became an unsafe environment for her. Relocation was previously considered together with social welfare officers when the situation became untenable, due to threats, violence, and when the cognitive decline made daily life unmanageable. In this case, the wife reported seeking help through the municipality and social services, yet the process stalled because it was a requirement for the individual with dementia to consent. This created a sense of powerlessness.

*“It was… the consequence of this behaviour. My son and I arranged a meeting with a social welfare officer from the municipality. They came*,* and they were very competent and helpful. But he refused. They explained about short-term care to give me relief. He refused… because he thought I was unfaithful*,* that I was with other men. So [he thought that]*,* it is I who must move*,* not him.”*

#### Barriers to admission to residential care

One of the greatest obstacles for admission to residential care, according to the wife, was his resistance to moving. This reluctance could be linked to identity, pride, and self-image, with relocation perceived as a sign of losing autonomy and status. Discussions about admission to residential care become sensitive and, at times, impossible.

A central concern from the wife was whether her husband would feel ‘at home’ in an unfamiliar environment in residential care. She described language and culture as crucial to the husband’s well-being and emphasised the importance of staff who could speak the husband’s native language and understand cultural references. This, she thought, together with the staff’s ability to see the person instead of the symptoms, was a key to creating safety and a sense of being recognised.

*“But he… aside from everything*,* he feels left out. He never listens to Swedish music. Why? That is my question. He speaks Swedish… yes… he speaks Swedish*,* but he is not engaged in Swedish culture. He does not want to. And then I think*,* ‘Oh my God… when he turns 80 and uses residential care*,* how will it be?’ Maybe he will start speaking his native tongue. Are there staff who speak his language? … Or English? What will it be like for him?”*

Music and small cultural elements were mentioned as important for the husband’s sense of familiarity. An admission to residential care represents an existential threshold, from a life built on independence to one where care needs dominate. For the wife, the transition in living conditions could bring relief, yet she describes it as mixed with guilt and grief. She saw the facility as an opportunity for respite and recovery, but also as a final stage in life’s journey.

*“And then*,* knowing that he is… in the right place… that he lives in the right environment*,* one that truly helps him. And for me*,* knowing he is in the right environment*,* that also makes me feel well.”*

## Discussion

This study revealed important cultural differences and similarities in how residential care is perceived in two cases of older immigrant families including one person with dementia and one relative, using interviews about their thoughts about taking part in official dementia care and about important factors when (and if) admission to residential care becomes necessary.

Regardless of the life situation, living together or being separated for safety reasons, residential care was consistently described as the end point of support strategies, rather than an initial or preferred form of assistance and relief for the unofficial caregiver. The results align with previous studies of Stenberg & Hjelm [5] that residential care is regarded as a very last resort for immigrants. This finding highlights cultural divergence. Sweden is one of the most individualised societies in the world [22], and care of people with dementia is organised around institutional solutions and residential care. However, residential care is costly for society and earlier in the dementia progression, outpatient care is offered by home health care services or in daycare as a complement to informal family carers. The two cases studied illustrate that this perception is linked to the perception of how everyday routines in dementia facilities are structured. Both cases emphasised the need of a holistic, person-centred approach which is based on the individual habits and wishes as well as being compliant to the symptoms of the disease and not merely designed to fit the workflow of the staff. Consequently, the interviews highlight the fear of the wives that the husbands are expected to adapt to predetermined patterns in residential care, rather than having care tailored to their unique needs, cultural background, and daily habits. These factors create a contradiction between the expectations of the wives and their own prior understanding of how residential care is provided. Knowledge of these contradictions is important for professionals meeting older immigrants who are planning to receive municipal care. Such meetings are important for building trust ahead of future care planning to be carried out by social welfare officers.

Such tensions underscore the importance and the ability to present a person-centred and culturally sensitive dementia care. For these immigrant families, the expectation was that care should preserve familiar routines, family involvement, and cultural practices for as long as possible. However, how these expectations differ from those of Swedish born people, is unknown. Residential care that is perceived as rigid and staff-centred could be experienced as alienating and misaligned with the values of relational and collective support. This is congruent with results of Cabote [4] which highlight specific needs among older adults with dementia in residential care settings, including culture-specific needs, such as shared language, traditional food, and spiritual or social practices, and also dementia-specific needs, such as a focus on comfort beyond clinical requirements and individualised care that considers behavioural symptoms. The results of this study are mostly similar to Cabote’s presented needs, except for the need for traditional food, which was not expressed in the interviews to the same extent as predicted [4]. This might be because the cases did not have a specific cultural connection to certain foods, or that the food habits are so ‘natural’ and basic for them that they did not could see that as a problem. Also, this could be a sign that both cases had adapted their cultural food habits towards traditional Swedish patterns. Recognising and adapting to these needs in residential care may depend on staff skills and competence in meeting individual cultural preferences, which indicates the need for key employees with specialised expertise in both cultural context and adaptation and in dementia care. The importance of key employees, and how their competence influences older adults’ perception of receiving good care, has been clearly described in previous research [23].

A lack of cultural competence among care staff, discriminatory attitudes, and inadequate organisational responses have been shown to further exacerbate the situation for these immigrant populations [3]. The need for healthcare and social services that are sensitive to issues related to immigration, culture and religion is highlighted by Lillekroken et al. [6]. Moreover, person-centred care reflecting Western ideals such as autonomy and individualism and which may not resonate with individuals from collectivist cultures where family involvement, spiritual beliefs, or community values, play a central role in decision-making [24, 25]. This cultural mismatch can lead to misunderstandings, reduced trust, and unmet needs, and also to situations in which persons with dementia postpone (or refuse) residential care, thereby resulting in inadequate care of the person with dementia. Such situations might also negatively affect health in the family member supporting and to increased total health care costs. Therefore, these issues must be addressed by stakeholders and policymakers to arise awareness and inform the general public, and more specific older immigrants, about the needs that can be met by residential care, as well as by home-based care.

Finally, the study reveals that admission to residential care is not merely a logistical decision but a human negotiation between safety, autonomy, cultural identity, and emotional bonds. Residential care thus, represents both a potential source of relief and a symbolic final stage in life’s journey, where the challenge lies in balancing professional support with the preservation of dignity and autonomy.

### Methodological considerations

The choice of a case study design [15] enabled an in-depth exploration of how older immigrants living with dementia, or caring for a spouse with dementia, think about taking part in official Swedish dementia care services, specifically residential care. With the two cases representing two couples consenting to participate, the study cannot claim to represent the wider population of older immigrants in Sweden. However, review studies of a larger population immigrants also show a complex picture of expectations and practices due to cultural values, traditions, and communication [6]. The participants represented in the cases were immigrants from different countries, with different native. They also had different cultural attitude towards caring for one’s relative and position on residential care and had different pre-understand of how the Swedish elderly care operates. Therefore, we would emphasise that the results cannot be interpreted as general for the immigrant population.Nevertheless, the detailed narratives provide valuable insights into culturally shaped perceptions of care and support, thereby strengthening the study’s contribution to understanding individual and cultural variation.

Recruitment proved to be particularly challenging, and this was also shown in similar target groups [16]. Despite outreach through municipal home care units, memory clinics, cultural organisations, and community networks, only two couples agreed to take part over a twelve-month period. This difficulty could reflect cultural understandings of dementia, which in some communities is perceived not as a medical condition but as a natural aspect of ageing, described as ‘forgetfulness’ [21], or lack of health literacy about dementia [6]. Previous research of older adults moving to a residential care [26] and immigrant relatives to people with dementia [16] has shown how declining to participate in a survey was due to 1) a lack of knowledge about dementia, as well as rules and regulations in Sweden, and 2) a sense of insecurity, based in previous experiences of unjust treatment, and 3) a cultural view of dementia affecting coping strategies of the disease and symptoms. In addition, family members often sought to protect older relatives from external scrutiny, which resulted in reluctance to share experiences with healthcare professionals or researchers. While the small sample size may be seen as a limitation, the recruitment challenges themselves highlight important structural and cultural barriers to undertaking research in this field, which is a significant finding. In future studies, a suggestion to get past this issue, could be to develop contact with a key person with a tight connection to the target group and perform recruiting with them in the key person’s forum for meetings. A key person in this context might be a host or a leader in a social environment where immigrants visit on regular basis, and who the visitors have great trust and confidence in. This could be a recruitment strategy to overcome difficulties in engage older migrant in future research.

Data collection through semi-structured interviews allowed participants to articulate their perspectives in their own words, while providing the researchers with the flexibility to probe further. As it was the wives who had no memory problems who were able to describe their situation in the greatest detail, which gave the interviews a deeper dimension, one suggestion for further research is to examine the experiences of immigrant wives in caring for their husbands. Conducting interviews in different settings, one in the participants’ home and the other in a separate room in a public library, may have influenced the dynamics of the conversations. The home environment may foster openness and comfort, whereas the neutral, public setting may encourage a more formal tone. These contextual differences should be considered when interpreting the findings.

The use of reflexive thematic analysis [17–19] facilitated a collaborative and dialogic approach to data interpretation. The two researchers brought distinct professional backgrounds: the first author, a registered dietitian with experience of engaging with individuals in similar life situations, and the second author, an occupational therapist with expertise in qualitative research and working with older adults. These different perspectives enriched the analysis but also introduced the possibility of bias through pre-existing assumptions. However, since the researchers had diverse backgrounds, any bias could be different and, accordingly the total risk of bias decreased. However, to strengthen credibility reflexive thematic discussions and joint coding sessions were therefore deemed essential to ensure transparency and to balance interpretation. Regarding dependability, the study followed established qualitative research guidelines [20] and obtained ethical approval, which may be interpreted as evidence of its dependability. The transparent description of recruitment challenges, interview settings, and analytic procedures provides a clear audit trail, allowing readers to understand how the findings were derived, and therefore possibilities for other interpretations. Further, ethical considerations were paramount, given the vulnerability of the participant group. Informed consent was obtained from all participants, which is a strength (and a given) in the study. However, the reluctance of many potential participants to engage, due to cultural perceptions of dementia, underscores the ethical complexity of conducting research in this area. In an ethical context, the interviews could also initiate a process of thoughts about these tough decisions about moving to residential care. The fact that expressing feelings might start a process of worrying about this transition, makes the interview itself an ethical dilemma, as the interviewers did not offer continued support. The interviews were planned to have plenty of time, thereby reducing any stress of the situation and having the possibilities to address feelings and worries.

## Conclusion

This study shows that despite differences in living arrangements and caregiving challenges, both cases described the complicated transition from home to residential care as an existential threshold based on barriers such as institutional routines, uncertain staff competence, cultural and linguistic mismatches, and the individual’s refusal to relocate. These barriers contribute to a fear of the personal needs of the person with dementia becoming solely a part of the routines of residential care, resulting in the loss of individual daily habits and personal ways of being. Stakeholders and policymakers must take this vulnerability into account when creating conditions for healthcare and social services aimed towards immigrants living with dementia.

## Supplementary Information

Supplementary Material 1.

## Data Availability

The datasets on which the conclusions in this article are based are not publicly available due to ethical and legal restrictions. Consequently, access to the data is available only upon request, following ethical approval from the Swedish Ethical Review Authority, that restricted access to the data.
