# Supplementary material for: Preserving togetherness or ensuring safety? The dilemma of where to live and receive dementia care
Source: BMC Geriatr. 2026 Jun 24;26:876. doi: 10.1186/s12877-026-07884-y (PMC13312748; doi:10.1186/s12877-026-07884-y)
Supplement: Supplementary file 1 — Supplementary Material 1. [file 12877_2026_7884_MOESM1_ESM.docx]

**Supplementary file – Interview guide**

Interview number ____ Date______________

**Interview Guide for Older Adults and Family Members, Independent Living**

Introduction before interview: We are interested in learning more about the experience of living with or caring for someone with cognitive impairment or memory loss. We would also like to know your thoughts on what we in Sweden refer to as residential care. Residential care are facilities you must apply to and be assessed as needing, for example, because daily life no longer functions due to impaired memory and the person needs to be close to others around the clock for help with all aspects of their personal care.

| **Demographic questions** | |
| --- | --- |
| What is your relationship with the person who has cognitive impairment or is forgetful? | |
| What is the person's cultural background? | |
| What language does this person speak? | |
|  | |
| I’m interested in learning more about what it’s like to grow older in Sweden when you come from a different cultural background. Right now, we’re particularly interested in hearing from people with cognitive impairment or dementia and their loved ones. | |
|  |  |
| **Interview Question 1** | **Follow-up questions** |
| 1. What are your general thoughts on growing older in Sweden? | Is it different from the countries where you’ve lived before? |
| 1. Can you tell us a little about what you know regarding residential care? | How did you come across that information?  Do you feel that information about such housing options is readily available?  How would you prefer to receive information about such housing options?  Is this a topic you discuss within your family?  Is there a tradition in your family of having close relatives living in such housing? |
| 1. What is important to you or your loved one if you or your loved one were to move into residential care? | If you imagine that someone close to you were to move into such a facility, what would you want for them?  What does that person need?  What do you, as a family, need? |
| 1. How would you feel about [letting your loved one] move into residential care if it became difficult for them to continue living at home due to their illness? What is your opinion based on? | Is there anything the residential care could do?  What does your family need? |
